# Supplementary material for: Lithosphere strain rate and stress field orientations near the Alpine arc in Switzerland
Source: Sci Rep. 2018 Jan 31;8:2018. doi: 10.1038/s41598-018-20253-z (PMC5792518; doi:10.1038/s41598-018-20253-z)
Supplement: Supplementary file 1 — Supplementary Figures [file 41598_2018_20253_MOESM1_ESM.doc]

**Supplementary Materials**

***"Lithosphere strain rate and stress field orientations near the Alpine arc in Switzerland"***

By Houlié, N.^1,2^ , Woessner, J. ^3,*^, Giardini, D.^1^ and Rothacher, M.^2^

^1^ ETH-Zurich, SEG, Sonneggstrasse 5, 8092 Zürich, Switzerland

^2^ ETH-Zurich, MPG, HPV G 53, Robert-Gnehm-Weg 15, 8093 Zürich, Switzerland

^3^ ETH-Zurich, SED, Sonneggstrasse 5, 8092 Zürich, Switzerland


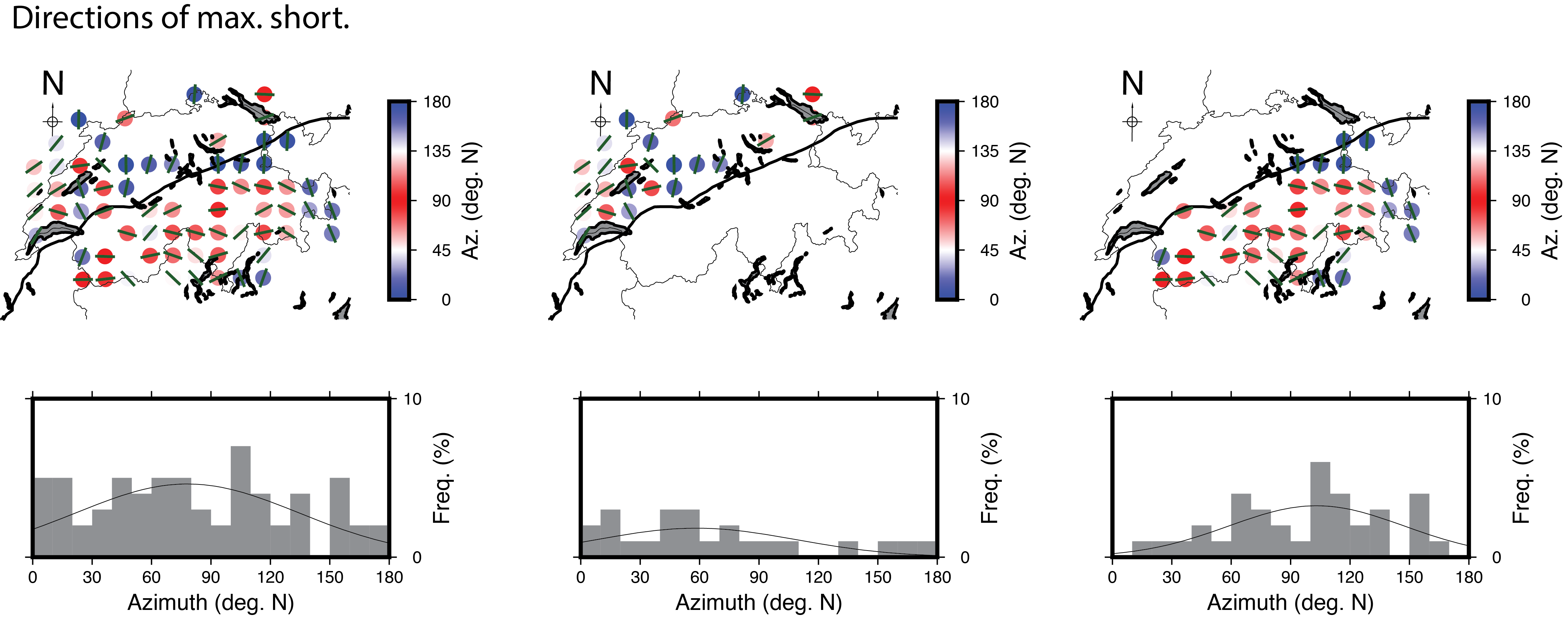


**Figure A1:** Directions of maximum shortening (GPS).


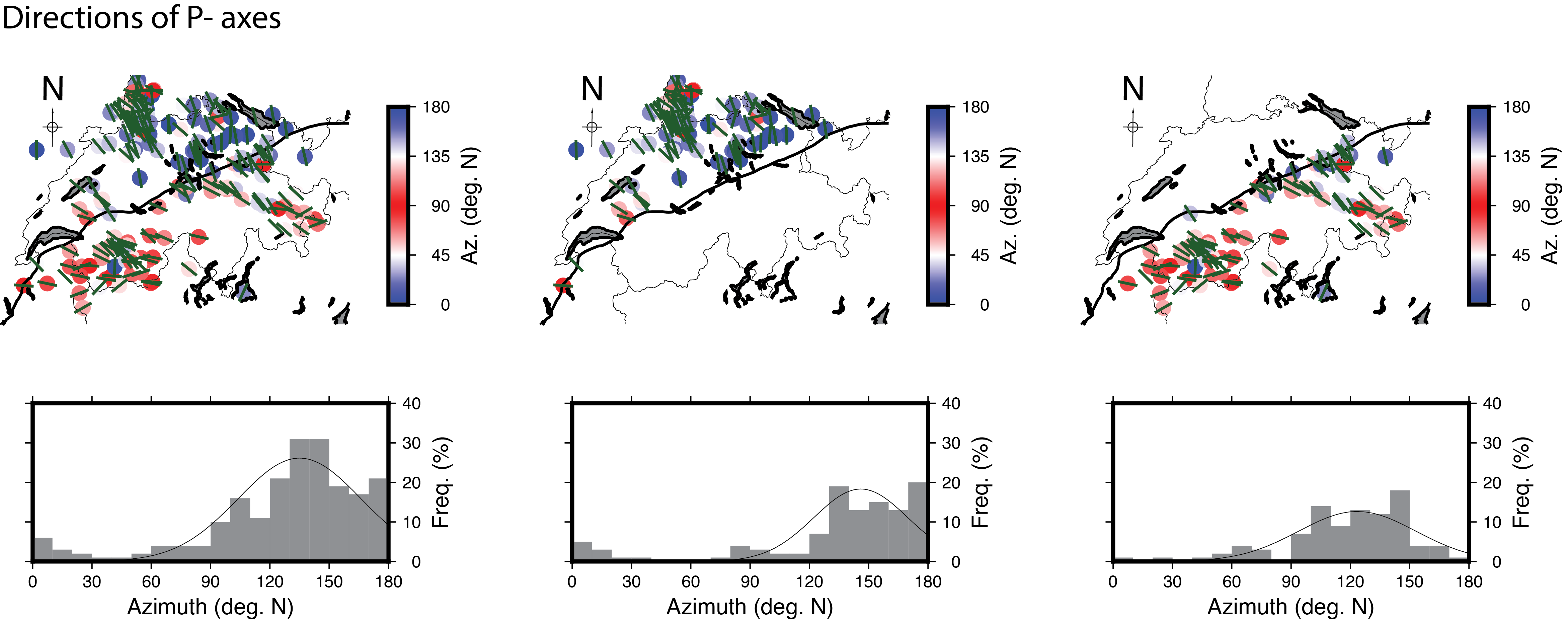


**Figure A2:** Directions of *P* axes (first motions)


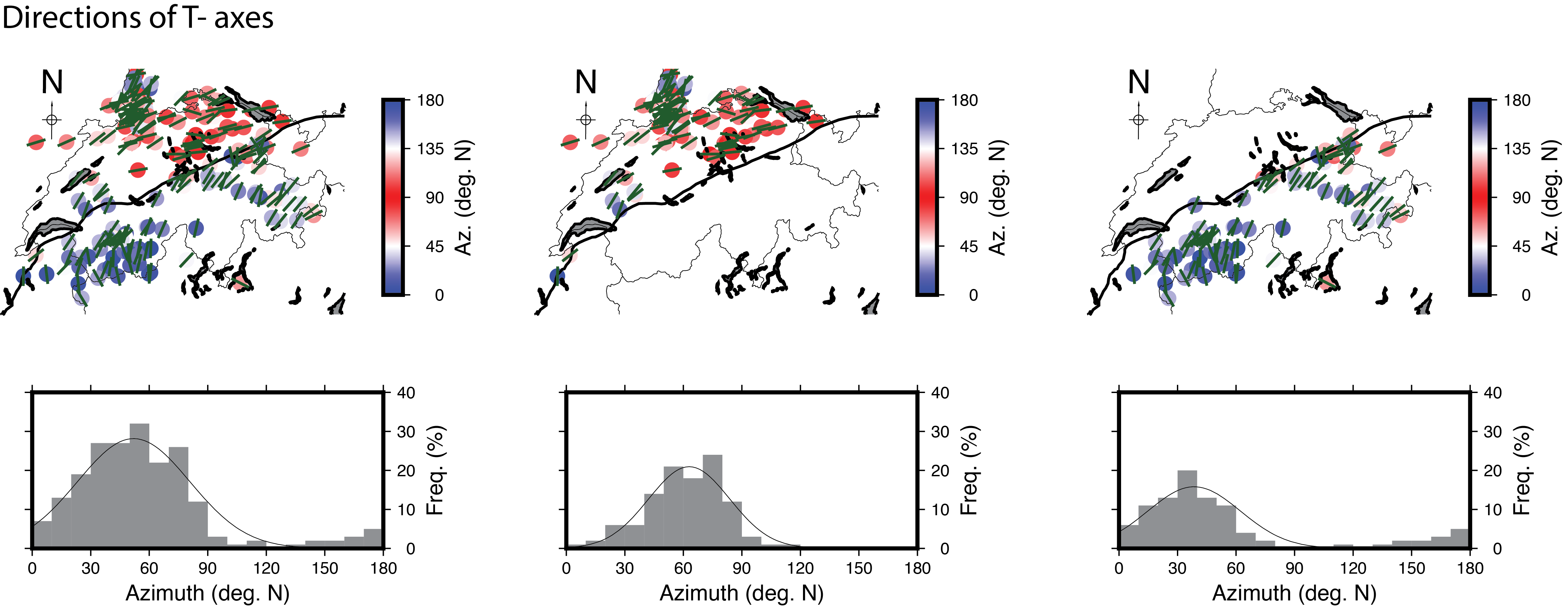


**Figure A3:** Directions of *T* axes (first motions)
